# Strategy to address innovative off-label medication use in China: grading management

**DOI:** 10.1007/s00228-014-1729-3

**Published:** 2014-08-17

**Authors:** Hanbin Wu, Gao Wu

**Affiliations:** 1 Clinical Pharmacy, Shanghai East Hospital, Tongji University School of Medicine, Shanghai, China; 2 Department of Pharmacy, The 411st Hospital of PLA, Shanghai, China

Dear Editor,

The off-label use of medication is an important part of medical practice worldwide. Off-label medication use enables clinicians practicing to become more knowledgeable about treatment alternatives. Off-label medication use may lead to innovative new uses of old medications and new therapeutic advances, particularly when approved treatments have failed. Off-label use of medication can vary from being experimental or innovative or controversial to standard practice and even state-of-the-art treatment. So off-label use of medication carries a higher risk for the patient and the practitioner than its registered use. Awareness remains an issue in many conditions for both patients and prescribers, but unfortunately, abuse and overpromotion continue too frequently, so extra care should be taken. For example, since more than 35 years, the international medical scientific community tries to solve the problem of the off-label use of pediatric drugs. But the detailed analysis of the problem shows not only a still remaining lack of medical knowledge, but also persistent weaknesses in the ethical, legal, medical, pharmacological, and political practices that surround the phenomenon of off label use in pediatrics [1]. So off-label use has become a worldwide problem. The key to solving this problem is how to regulate off-label promotion. Off-label prescribing is legal in the USA and in many other countries. Zhang et al. reported in their study that seven countries had laws related to off-label medication use: American, Germany, Italy, Netherland, New Zealand, India, and Japan. Except India [2], the rational off-label medication use was allowed in the other six countries. The right to prescribe off-label drug was defined in Britain and Ireland. Ten countries published guidelines or statements related to off-label medication use by their official departments and academic organizations [3]. According to FDA regulations, physicians may prescribe drugs for off-label use, but drug manufacturers may not promote such uses [4]. The off-label prescriptions must better serve patient needs than alternatives and must be supported by evidence or experience to demonstrate safety and efficacy in the UK. But the responsibility for regulating and monitoring use of innovative off-label medications rests at a local level. Medical institutions must have an institutional policy to govern innovative off-label medication use. The institutional policy may be more efficient to focus on the medications that effective and risk, rather than attempting to control the use of all medications prescribed innovative off-label use. Ansani et al. had designed a strategy to promote safe, innovative off-label use of medications that was a formal, approved, systematic approach for structured therapeutic guideline recommendations for innovative off-label drug use. This approach includes crafting supporting documents, grading the level of scientific evidence, and incorporating patient safety monitoring into medication review [5].

Off-label medication use is common practice in China. Our study shows that the off-label drugs percentage in therapeutic drugs during hospitalization was 47.64 %, most case lacked evidence of clinical efficacy [6]. Compared with the USA, China law does not currently specify whether off-label medication use is illegal. Ministry of Health, Measures for the Administration of Prescriptions Chapter 4, Section 14 provides that “In the description of the drug, physicians should be based on medical, prevention, health care needs, according to the diagnostic and treatment practices, and drug indications, pharmacological effects, usage, dosage, contraindications, adverse reactions and precautions in the package insert.” This means that prescribing of approved drugs must be based on the diagnostic and treatment practices and/or on the package insert. So there are no standardized regulations on off-label use in China. Despite the widespread recognition of the problem, few institutions have a mechanism to control innovative off-label medication use in China. Medical institutions must take the responsibility for regulating and monitoring use of innovative off-label medications. Therefore, a management guideline for off-label medication use is urgently needed in medical institution. Ideally, any off-label medication use should be in the context of a study in which informed consent is given and approval by an Institutional Review Board is obtained. This article describes a strategy, a systematic approach to innovative off-label medication prescribing and provides a mechanism for the evolution and promotion of standards of medical care for this situation, ensure oversight of patient safety, and prospectively assess efficacy.

We convened a multidisciplinary ad hoc task force (Institutional Review Board) to actively address innovative off-label medication use and to define optimal medication management through the hospital’s Pharmacotherapy, which traditionally have been responsible for promoting safe, effective medication use and making recommendations for drug use through formulary review. The committee should be considered the arbiter of off-label use, should develop therapeutic guideline for innovative off-label use of an approved drug. To maintain focus on the underlying evidence, a grading management system was developed, which grade the quality of the evidence of off-label medication use and to evaluate the strength of recommendation of the intervention that is proposed in the information of off-label medication use. The system looks at four factors to determine the quality of the evidence: study design (the number and size of clinical trials,), study quality (study design and conduct), consistency, and directness (or generalizability). After combining the four components and assessing the grade of the evidence, the strength of recommendation of the intervention is established. The practicing clinicians use them in making safe and rational formulary decisions about patient care. Generally, all available data and information would be collected, assessed, and analyzed by applying a ranked grading system. The elements required by policy within this written proposal must incorporate an executive summary, rationale, background, evidence support, appropriate selection criteria for patients, instructions for clinical conduct (including dose, duration, and regimen), clinical efficacy and safety outcomes, patient informed consent, a proposed timeline for evaluation (with a maximum limit of a year), and pharmacoeconomic outcomes. Providing information should include approved labeling, comprehensive bibliography of publications related to off-label use and other available information about risks of this use, plus representative publications reaching conclusions regarding this use including contrary or different statement. Further, the following principles should guide the innovative off-label use of medications:

1. Patient safety should be the primary consideration.
2. Physicians may also need to self-monitor their prescribing practices in order to avoid inappropriate.
3. Off-label prescribing should be meet ethical obligations to the patient and society.
4. The ultimate responsibility for the safety and efficacy of off-label use resides with the prescriber.
5. The hospital’s Pharmacotherapy Committee offers expedited passport services to urgent patient care issues for off-label prescribing.

The hospital’s Pharmacotherapy Committees has established five categories in the standardized formulary review to indicate the conditions under which information about the off-label uses of drugs to adjudicate conflict between physicians, patient, and/or pharmacy in the initial evaluation or subsequent interim analysis of new information, by which based on the grading management system to evaluate the methodological rigor of clinical data. The categories are the following:

Category A The off-label medication use, in the same manner as it would be for an on-label use grounded by scientific evidence, is inconsistent with present labeling, but that do the “Chinese National Formulary,” medical textbooks, the latest clinical practice guidelines, clinical pathways, and other authoritative information on expert consensus support. In this case, it is consistent with the policy of Measures for the Administration of Prescriptions that the physicians prescribe therapies according to “the diagnostic and treatment practices.” The formulary review is similar to process of approved medication use. For example, propranolol can be used migraine headache treatment in “Chinese National Formulary,” which is unapproved in present labeling [7]. There are no restrictions in the situation. We call this conversation routine therapeutic consent.
Category B The off-label medication use may originate from a presumed drug class effect. This approval grants formulary addition and is based on the judgment that adequate evidence exists for an off-label formulary addition. It requires more discussion than routine therapeutic consent but less formality than written informed consent. There is requirement of double signatory by the physician on the prescription in the formulary review. We call this type of consent augmented therapeutic consent. For example, the use of propranolol for portal hypertension could reduce portal pressure that the pharmacological effect is an extension of splanchnic vasoconstriction of propranolol.
Category C The off-label use medication has a reasonable therapeutic rationale but here is insufficient evidence to thoroughly allay concerns about its safety, efficacy, and cost-effectiveness for the innovative use, yet it is not considered a clinical research. This category restricts drug use within a “therapeutic guideline for innovative off label use of an approved drug,” and require rigorous informed consent for the situation whereby patients are informed of potential risks and benefits and understand that the clinical outcome of this medication use is uncertain prior to administering the drug. Upon review by the Board, the Committee must again review for a final vote of formulary status. For example, the therapeutic guideline of propranolol for infantile hemangioma must be approved to formulary by the hospital’s Pharmacotherapy Committee. We call this therapeutic guideline approved therapeutic consent.
Category D The off-label use medication has little or no supporting evidence. The evidence provides information only about cases. The off-label use (with very low certainty of net benefit) for the indication is restricted to clinical research (is considered experimental). Evidence of potential harms of the off-label use should be sought out and considered at least as inclusively and rigorously as the evidence for potential benefit. The off-label use generally should be limited to the context of research protocols. Patients should engage in a formal informed consent process with the physician researcher before receiving drugs for experimental off-label use. Patients must understand that the purpose of experimental off-label use is to generate data to improve medical care for future patients rather than to provide medically indicated treatment, although the patient may benefit. An application for off-label use medication should be submitted and approved by the hospital’s Pharmacotherapy Committee and hospital’s Ethics Committee. We call this therapeutic approach specified therapeutic consent. For example, the application of propranolol to treat anxiety disorders must be submitted and approved.
Category X There is evidence that benefit of the off-label use medication is unfavorable, and the risks involved in off-label use of the medication in the indication clearly outweigh potential benefits. The proposed clinical use of a medication is considered inappropriate, and the medication use in the requested manner is not permitted by the hospital’s Pharmacotherapy Committee.

More and more people recognized that public health might be advanced if health-care professionals were to receive information about the off-label use that is truthful and not misleading. But busy physicians would not have the time to do their own due diligence regarding off label uses; therefore, there is a need for informing physicians about the evidence supporting off-label prescriptions. Before considering off-label use, supporting safety and efficacy evidence must be carefully evaluated and a risk–benefit determination made, especially when alternatives with approved labeling are available for the intended off-label use. Our strategy that involves review of all off-label uses of medications by formulary review and input from expert panels develops a comprehensive and workable ethical framework that prioritizes scrutiny of off label prescribing for practicing physicians. This addresses issues related to off-label prescribing at a specific institutional level. By classifying off-label uses as five categories, this practical strategy grounds recommendations for prescribing practices in a judgment of the strength of the evidence for net health benefit. This modifies innovative off-label medication use decisions as well as contributes to expanding clinical information bases. Such restraint not only protects patients from unsafe or ineffective off-label uses but also guards physicians against tort liability and medical malpractice suits.
